# Acetaminophen impairs ferroptosis in the hippocampus of septic mice by regulating glutathione peroxidase 4 and ferroptosis suppressor protein 1 pathways

**DOI:** 10.1002/brb3.3145

**Published:** 2023-07-13

**Authors:** Jing Chu, Hong Li, Zhihao Yuan, Wenyu Zhou, Yang Yu, Yonghao Yu

**Affiliations:** ^1^ Department of Anesthesiology Characteristic Medical Center of Chinese People's Armed Police Force (PAP) Tianjin China; ^2^ Department of Anesthesiology Tianjin Medical University General Hospital Tianjin China; ^3^ Tianjin Institute of Anesthesiology Tianjin China; ^4^ Department of Anesthesiology Tianjin Children's Hospital Tianjin China

**Keywords:** acetaminophen, ferroptosis, FSP1, GPX4, sepsis‐associated encephalopathy

## Abstract

**Background:**

Neuronal ferroptosis is a major cause of cognitive impairment and mortality in patients with sepsis‐associated encephalopathy (SAE). A low dose of acetaminophen (APAP) in septic mice can prevent ferroptosis in the hippocampal tissue; however, the underlying mechanism is unknown. This study aimed to investigate the mechanism by which APAP reduces ferroptosis in the hippocampal tissues of septic mice.

**Methods:**

A mouse model of SAE was established, and the ferroptosis pathway inhibitors RSL3 and iFSP1+RSL3 were used in addition to APAP for the interventions, respectively. The 7‐day survival rate of the mice was recorded, and cognitive function was examined using the Morris water maze test. Hematoxylin and eosin staining was performed to observe hippocampal tissue damage. Hippocampal iron and malondialdehyde (MDA) were measured using chemical colorimetric methods. Immunofluorescence was used to detect the reactive oxygen species (ROS) content in hippocampal tissues.

**Results:**

RSL3 reversed the efficacy of APAP on improving cognitive dysfunction in septic mice but did not obviously reverse the survival rate of mice enhanced by APAP. RSL3 aggravated APAP‐induced hippocampal tissue damage in mice attenuated by APAP. RSL3 inhibited glutathione peroxidase 4 (GPX4) expression and increased ferroptosis suppressor protein 1 (FSP1) and 4‐hydroxy‐2‐nonenal (4‐HNE) expression. RSL3 also reversed the effects of APAP in reducing iron, MDA, and ROS levels in the hippocampal tissues of septic mice. iFSP1+RSL3 further reversed the effect of APAP on ameliorating cognitive dysfunction in septic mice and successfully reversed the survival rate of mice enhanced by APAP. iFSP1+RSL3 aggravated APAP‐induced cerebral hippocampal damage. iFSP1+RSL3 inhibited both GPX4 and FSP1, further reversing the effect of APAP on the reduction in iron, 4‐HNE, ROS, and MDA levels in the cerebral hippocampus of mice with sepsis.

**Conclusion:**

These data suggest that APAP inhibits ferroptosis in the cerebral hippocampus of septic mice through the GPX4 and FSP1 pathways.

## Graphical abstract

Neuronal ferroptosis is a major cause of cognitive impairment and mortality in sepsis‐associated encephalopathy (SAE). In septic mice, a little dosage of acetaminophen (APAP) can prevent hippocampus tissue ferroptosis, however the mechanism is unknown. The goal of this study was to investigate the mechanism through which APAP reduces ferroptosis in septic mice hippocampus tissue.

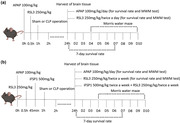

## 1 INTRODUCTION

Sepsis‐associated encephalopathy (SAE) is a broad cerebral dysfunction caused by sepsis. Patients seldom develop central nervous system infections. In individuals with sepsis, the prevalence of SAE varies from 9% to 70% (Wei et al., 2022). In patients with sepsis exacerbated by abrupt changes in mental status, mortality in critical care units can reach 49% (Gao et al., 2022). Acetaminophen (APAP) at low dosages protects neurons from oxidative stress (Locke et al., 2008; Maharaj et al., 2004). According to our previous findings, APAP reduced hippocampal tissue ferroptosis in septic mice and improved cognition and survival (Chu et al., 2022). However, the mechanism by which APAP inhibits ferroptosis in mice with sepsis remains unclear.

The two major ferroptosis‐negative regulatory mechanisms identified thus far are glutathione peroxidase 4 (GPX4) and ferroptosis suppressor protein 1 (FSP1). Early research revealed that low doses of APAP produced neuroprotective benefits via nonenzymatic antioxidant mechanisms, including the regulation of glutathione peroxidase (GSH‐Px) enzyme activity and vitamin E (α‐tocopherol) levels (Ghanem et al., 2016). This is intimately connected to the primary ferroptosis pathways GPX4 and FSP1.GPX4 is the main role In GSH‐Px, FSP1 indirectly suppresses ferroptosis via vitamin E. In the present study, we suggest that APAP inhibits ferroptosis in mice with sepsis via the GPX4 and FSP1 pathways. GPX4 and FSP1 pathway inhibitors were used to gain insight into the molecular mechanisms of APAP‐induced ferroptosis suppression.

## 2 MATERIALS AND METHODS

### 2.1 Animals

Male C57BL/6J mice (age: 6–8 months old; weight: 20–24 g) were purchased from Co. (Tianjin, China). Five mice were housed per cage with a temperature of 18–25°C, humidity of 25%−75%, and a 12 h light‐dark cycle. Food and water were provided to the mice when needed. All the experimental protocols were approved by the Institutional Animal Care and Use Committee of the General Hospital of Tianjin Medical University (approval no. IRB2020‐DW‐07).

### 2.2 Cecal ligation and puncture model

The cecal ligation and puncture (CLP) model was constructed according to the method of a previous study (Drechsler & Osuchowski, 2021). Briefly, 50 mg/kg sodium pentobarbital solution was used to anesthetize the mice. Then, they were placed in a supine position, sterilized, and the skin was incised with ophthalmic scissors; a 1 cm incision was made along the midline of the abdomen. When the cecum was exposed, we ligated it under the ileocecal flap, followed by a puncture with a 20‐gauge needle, and squeezed out approximately 0.3 mL of intestinal contents. The cecum was returned to the abdominal cavity, and the peritoneum and skin were sutured layer by layer. In addition, primaxin (0.5 mg) dissolved in 200 μL sterile saline was injected immediately into each mouse after the procedure.

### 2.3 Experimental design

#### 2.3.1 Dose screening experiments

To understand the effect of different doses of RSL3 on the survival rate of mice, the mice were divided into five groups according to the random number table method (*n* = 20): control group (CON group), R50 group (50 mg/kg RSL3), R100 group (100 mg/kg RSL3), R250 group (250 mg/kg RSL3), and R500 group (500 mg/kg RSL3). The RSL3 was dissolved in 0.2 mL of saline and injected intraperitoneally into the mice. In contrast, the CON group was injected intraperitoneally with 0.2 mL of saline. All groups continued to be injected intraperitoneally twice a week until the end of the experiment. Survival was recorded for all mice within 7 days.

To understand the impact of different doses of iFSP1 on the survival rate of mice, the mice were divided into five groups according to the random number table method (*n* = 20): control group (CON group), iF50 group (50 mg/kg iFSP1), iF100 group (100 mg/kg iFSP1), iF250 group (250 mg/kg iFSP1), and iF500 group (500 mg/kg iFSP1). iFSP1 was dissolved in 0.2 mL of saline and injected intraperitoneally into the mice. In contrast, the CON group was injected intraperitoneally with 0.2 mL of saline. All groups continued to be injected intraperitoneally twice a week until the end of the experiment. Survival was recorded for all mice within 7 days.

To understand the impact of different doses of iFSP1 combined with RSL3 on the survival rate of mice, the mice were divided into six groups according to the random number table method (*n* = 20): control (CON), iF50+RSL3 (50 mg/kg iFSP1+250 mg/kg RSL3), iF100+RSL3 (100 mg/kg iFSP1+250 mg/kg RSL3), iF250+RSL3 (250 mg/kg iFSP1+250 mg/kg RSL3), and iF500+RSL3 group (500 mg/kg iFSP1+250 mg/kg RSL3). iFSP1 and RSL3 were dissolved in 0.2 mL of saline and injected intraperitoneally into mice at 30 min intervals twice a week until the end of the experiment. Survival was recorded for all mice within 7 days.

#### 2.3.2 Intervention experiments

Based on the results of the dose screening experiments, 250 mg/kg RSL3 or 250 mg/kg RSL3+500 mg/kg iFSP1 were used to verify whether APAP exerts its ferroptosis inhibitory effect through the GPX4 pathway or GPX4+FSP1 pathway.

**FIGURE 1:**
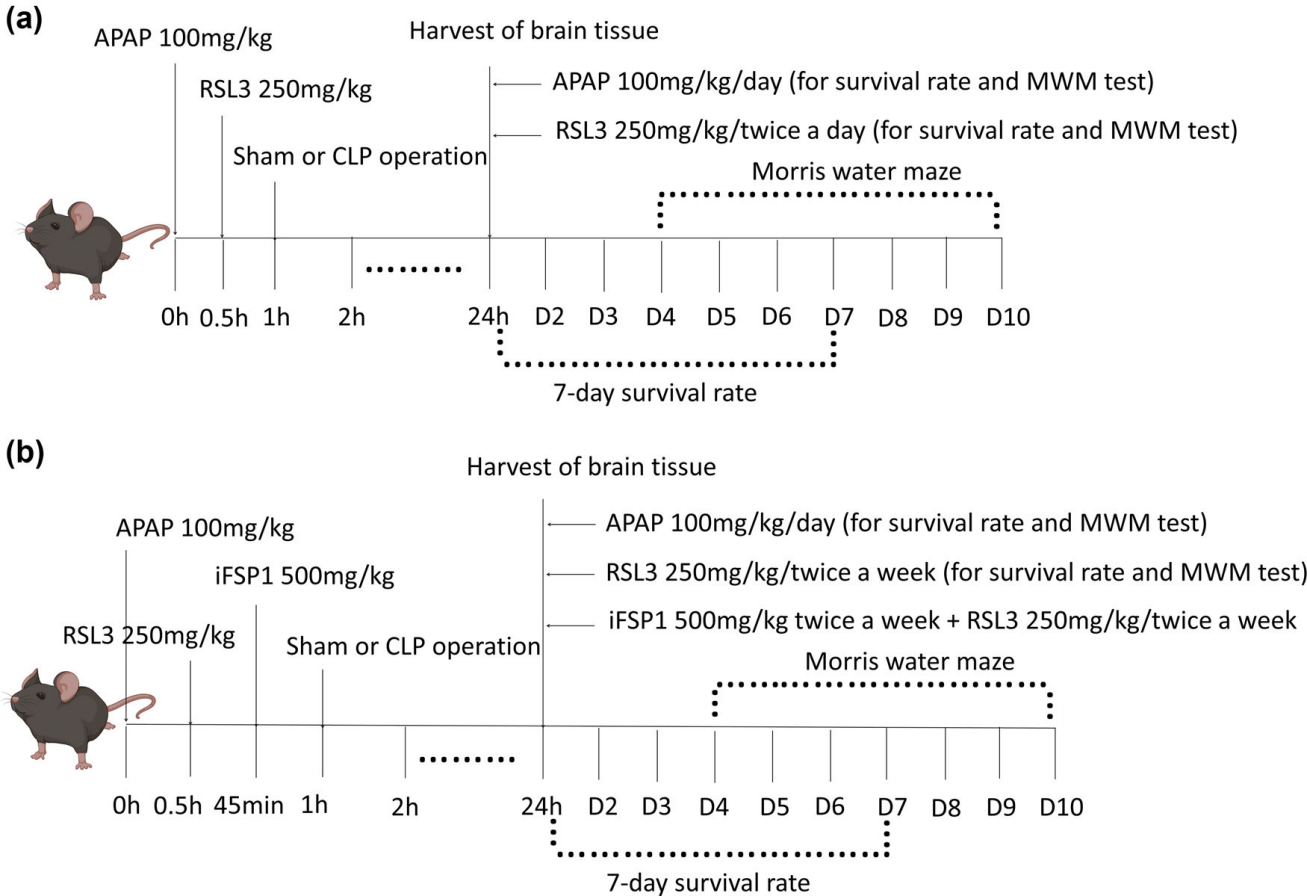
Experimental design. (A) 100 mg/kg APAP and/or 250 mg/kg RSL3 or an equal volume of normal saline were injected intraperitoneally before the sham or CLP operation, respectively, in male C57BL/6J mice. APAP 100 mg/kg or an equal volume of normal saline were injected 1 h before the sham or CLP operation and were injected per day continuously until the end of the test. Then, 250 mg/kg RSL3 or an equal volume of normal saline were injected 30 min before the sham or CLP operation and were injected twice a week until the end of the test. The brain tissues of different groups were harvested for tests 24 h after the sham or CLP operation. The Morris water task was conducted from days 4 to 10 after the sham or CLP operation. (B) 100 mg/kg APAP and/or 250 mg/kg RSL3 and/or 500 mg/kg iFSP1 or an equal volume of normal saline were injected intraperitoneally before the CLP operation, respectively, in male C57BL/6J mice. APAP 100 mg/kg or an equal volume of normal saline were injected 1 h before the CLP operation and were injected per day continuously until the end of the test. 250 mg/kg RSL3 or an equal volume of normal saline were injected 30 min before the CLP operation and were injected twice a week until the end of the test. 500 mg/kg iFSP1 or an equal volume of normal saline were injected 45 min before the operation and were injected twice a week until the end of the test. An equal volume of normal saline was injected at the same time before the sham operation for mice in the control group. The brain tissues of the different groups were harvested for tests 24 h after the sham or CLP operation. The Morris water test was conducted from days 4 to 10 after the sham or CLP operation. Abbreviations: APAP, acetaminophen; CLP, cecal ligation and puncture; iFSP1, an FSP1 inhibitor; RSL3, a GPX4 inhibitor.

To explore the GPX4 pathway, the mice were randomly split into six groups: sham, sham+APAP, CLP, APAP+CLP, RSL3+CLP, and APAP+RSL3+CLP. Mice in the CLP, APAP+CLP, RSL3+CLP, and APAP+RSL3+CLP groups underwent CLP surgery, whereas mice in the sham and sham+APAP groups underwent sham surgery (only the cecum was explored and then returned without ligation of the perforation). Then, 100 mg/kg of APAP was injected intraperitoneally 1 h before surgery for mice in the sham+APAP, APAP+CLP, and APAP+RSL3+CLP groups. Meanwhile, mice in the sham, CLP, and RSL3+CLP groups were injected intraperitoneally with 0.2 mL of saline, and all continued to be injected intraperitoneally once a day until the end of the experiment. The CLP group and APAP+RSL3+CLP group were injected intraperitoneally with 250 mg/kg of RSL3 0.5 h before surgery, and the mice in the remaining groups were given the same volume of saline. All mice were injected intraperitoneally twice a week until the end of the experiment (Figure 1A).

To explore the FSP1+GPX4 pathway, the mice were randomly split into four groups: sham, APAP+CLP, APAP+RSL3+CLP, and APAP+RSL3+iFSP1+CLP. Mice in the APAP+CLP, APAP+RSL3+CLP, and APAP+RSL3+iFSP1+CLP groups underwent CLP surgery, and 100 mg/kg of APAP was injected intraperitoneally 1 h before surgery. The sham group underwent sham surgery and was administered the same volume of saline, and all continued to be injected intraperitoneally once a day until the end of the experiment. Mice in the APAP+RSL3+CLP and APAP+RSL3+iFSP1+CLP groups were injected intraperitoneally with 250 mg/kg of RSL3 0.5 h before surgery, while the mice in the other groups were injected with the same volume of saline. Mice in the APAP+RSL3+iFSP1+CLP group were injected intraperitoneally with 500 mg/kg of iFSP1 45 min before surgery, whereas mice in the other groups were administered the same volume of saline and injected intraperitoneally twice a week until the end of the experiment (Figure 1B).

The same assays were adopted for both parts of the experiment as follows. Some mice in each group were sacrificed and infused 24 h after their operation to harvest their brain tissue. Other mice (*n* = 20 per group) were selected to evaluate their 7‐day survival status. At 4–10 days after their operation, the mice underwent the Morris water maze (MWM) test (*n* = 10 per group). GPX4, FSP1, and 4‐HNE protein levels in hippocampal tissue were detected by western blotting (*n* = 6 per group). Chemical colorimetry was used to detect iron and malondialdehyde (MDA) levels, and immunofluorescence was used to detect reactive oxygen species (ROS). Hematoxylin and eosin (HE) staining of the brain sections (*n* = 3 per group) was performed to observe hippocampal tissue damage.

### 2.4 Drug configuration

APAP configuration: APAP (Cat#B3535, APExBIO) 100 mg/kg was melted in 0.2 mL of normal saline. The dosage of APAP used was selected based on our previous use of its dose for SAE (Chu et al., 2022).

To configure RSL3, 500 mg of RSL3 (Cat# HY‐100218A, MCE) was added to 2.268 mL of dimethylsulfoxide (DMSO) and sonicated to aid solubilization. Next, DMSO (10%), PEG300 (40%), Tween‐80 (5%), and saline (45%) were added sequentially to the prepared solution and sonicated to aid solubilization. Finally, the solution was diluted according to the experimental requirements. Different doses of RSL3 were dissolved in 0.2 mL of saline.

To configure iFSP1, 250 mg of iFSP1 (Cat# HY‐136057, MCE) was added to 3.093 mL of DMSO and sonicated to aid solubilization. Next, DMSO (10%), PEG300 (40%), Tween‐80 (5%), and saline (45%) were added sequentially to the prepared solution and sonicated to aid solubilization. Finally, the solution was diluted according to the experimental requirements. Different concentrations of iFSP1 were dissolved in 0.2 mL of saline.

### 2.5 Survival rates

The survival rate was recorded within 7 days after the CLP or sham surgery.

### 2.6 MWM test

MWM experiments were conducted based on a previous study (Othman et al., 2022) to detect altered cognitive function in various groups of mice. The MWM system consisted of a black plastic circular pool (height 50 cm; diameter 100 cm) with different symbol shapes on its inner walls. A platform (diameter, 6 cm) was placed 1 cm underwater in one of the four quadrants. On days 4–10 after surgery, mice in each group underwent hidden platform exploration experiments. Mice were placed in each quadrant to find the hidden platform. Escape and swimming speeds were recorded. The escape latency was marked as 90 s for the mice that could not locate the hidden platform within 90 s.

On day 10, 2 h after the end of the experiment, the platform was removed, and an exploratory experiment was conducted. The experiment was stopped after each mouse had swum in the water for 60 s. The number of platform crossings, time spent in the target quadrant, and the number of platform crossings were recorded using a digital camera to demonstrate memory capacity.

### 2.7 HE staining

The mouse brain sections were soaked in paraformaldehyde (4%) for 24 h. After dehydration, the sections were embedded in paraffin, sectioned, and stained with HE. Morphological structural changes in the CA1 region of the hippocampus were observed and analyzed by microscopy. HE staining of brain sections was performed to observe hippocampal tissue damage using microscopy (Leica Microsystems).

### 2.8 Iron and MDA assay (colorimetric)

Hippocampal tissues were taken at 24 h post‐mold, and the iron (Cat# MAK025, Sigma) and MDA (Cat# MAK085, Sigma) contents were detected according to the manufacturer's instructions.

### 2.9 Western blotting

GPX4, FSP1, and 4‐HNE levels in hippocampal tissues were measured using western blotting. Briefly, hippocampal tissues were lysed, homogenized, and centrifuged to obtain supernatants, after which the BCA proteins were quantified and denatured by boiling after adding buffer. The resulting samples were processed using SDS‐PAGE, shifted to nitrocellulose membranes, then incubated in skim milk (5%). Then, the following primary antibodies were detected: GPX4 (Cat# SAB5700944, Sigma), FSP1 (Cat# SAB5701199, Sigma), 4‐HNE (Cat#ab46545, Abcam), and β‐actin (1:2000, Cat#ab5494, Abcam). After flushing, the samples were incubated overnight at 4°C. Goat anti‐rabbit antibody was added (1:4000, Cat#AP132, Sigma), and the samples were incubated for 1 h at 37°C. Nitrocellulose membranes were rinsed with TBST and treated with an enhanced chemiluminescence assay kit (Cat#ab65623, Abcam). The density of the strips was scanned, photographed, and analyzed using a Bio‐Rad Image Analysis System. The ratio of the strip grayscale value to the β‐actin grayscale value is considered to reflect the expression level of the target protein.

### 2.10 ROS assay

An immunofluorescence assay was used to evaluate the ROS levels in the hippocampal tissue. Moreover, 24 h after CLP or sham surgery, the mice were deeply anesthetized and perfused with phosphate‐buffered saline (PBS) using the transcardial perfusion method to obtain bloodless brains. Fresh brain tissues were placed in a −20°C refrigerator and processed on ice for 30 min, after which the frozen brain tissues were sectioned to 10 μm thicknesses and incubated with a superoxide anion fluorescent probe dihydroethidium (Cat#HY‐D0079, MCE) at 37°C for 1 h. Staining was followed by three washes with PBS containing Tween (PBST) for 5 min. For nuclear staining, the slices were stained with diamidino phenyl indole for 1–2 h before blocking and then washed three times with PBST for 5 min. The slides were sealed with an antifluorescence‐attenuating sealer. The slides were scanned using a fluorescence microscope (Biorevo). Hippocampal tissue sections of the mice were observed and photographed, and red fluorescence intensity was recorded and analyzed. Quantitative analysis of the ROS levels within the hippocampal tissue in each group of mice is shown as the ratio of its fluorescence intensity to that of the sham group. The ROS level of the sham group was defined as 100%.

### 2.11 Statistical analysis

Survival rates are expressed as percentages (%); escape latency, time spent in the target quadrant, and swimming speed in the MWM experiment are expressed as the mean ± standard deviation (SD). The number of platform crossings was expressed as the median and interquartile range. Other biochemical data are expressed as the mean ± SD. The Bonferroni‐corrected log‐rank (Mantel−Cox) test was used to analyze the differences in group survival rates. The Mann−Whitney U test was used to evaluate differences in the number of platform crossings for mice in each group. Escape latency and swimming speed in the MWM were analyzed using a two‐way repeated measures analysis of variance (ANOVA). Other behavioral experimental data were compared between the groups using a one‐way ANOVA. A one‐way ANOVA was used to compare other biochemical data between groups. Statistical significance was set at *p* <0 .05, and significance tests were two‐tailed. Statistical analyses were performed using GraphPad Prism software (version 8.0) and SPSS (version 22.0).

## 3 RESULTS

### 3.1 RSL3 reverses the inhibitory effect of APAP on ferroptosis in the cerebral hippocampus of septic mice

HE staining of brain sections was performed 24 h after surgery. Disorganized neuronal arrangements, deep cytoplasmic staining, nuclear consolidation, and structural obscurity were observed in the CA1 region of the hippocampus, indicating that the SAE mouse model was successfully modeled.

**FIGURE 2:**
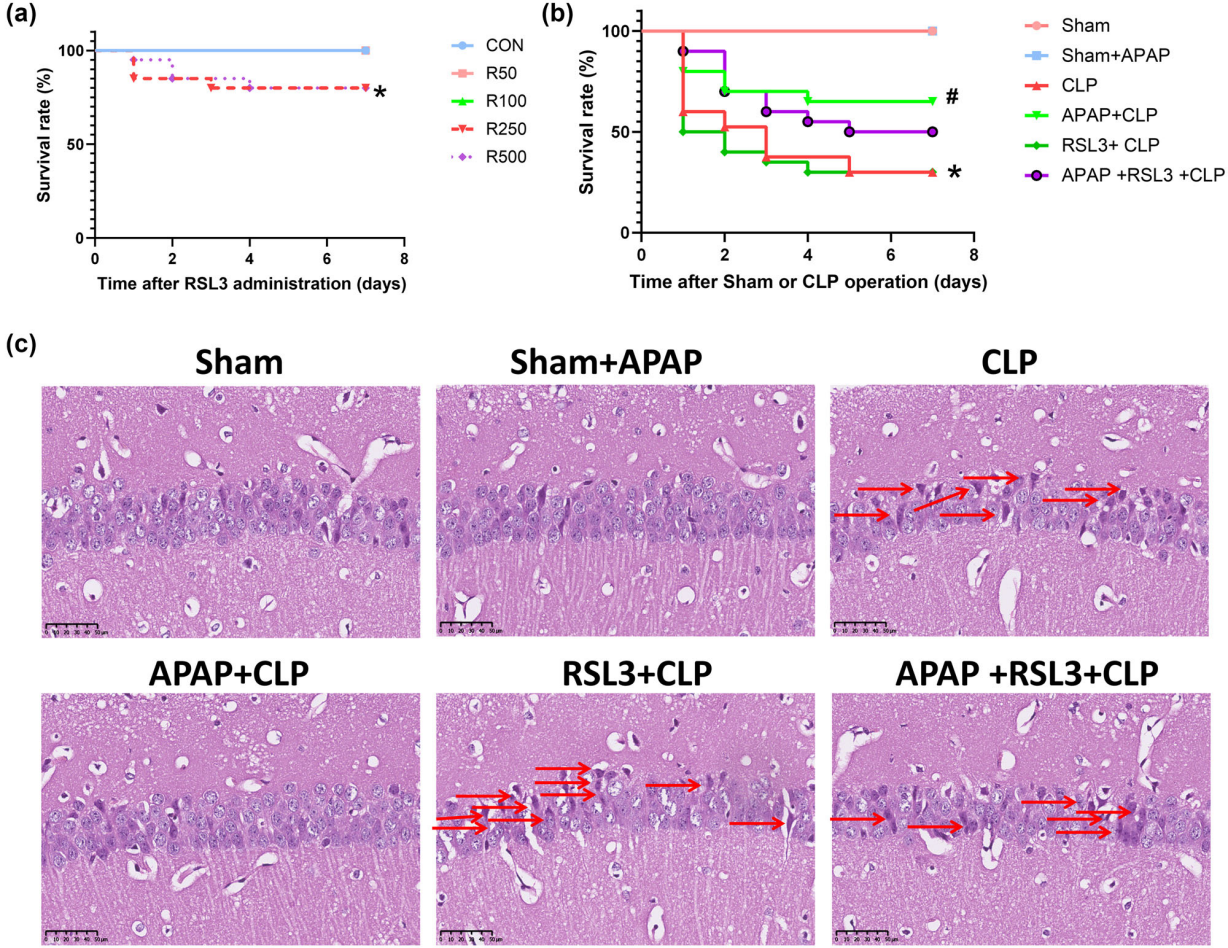
Effect of different doses of RSL3 on the survival rate of healthy mice (A). Effect of RSL3 on the survival rate of septic mice; APAP increased the survival rate of septic mice, which was not revered by RSL3 (B). RSL3 reverses the improved hippocampi tissue damage of septic mice with APAP treatment (C). (A, B) Values are depicted as survival percentages (*n* = 20). *p < 0.05 versus CON group (A). *p <0 .05 versus sham group; ^#^ *p* <0 .05 versus CLP group (B). (C) Brain tissue sections were made and stained with hematoxylin and eosin. The hippocampi structure in the CA1 region was observed.

This study is the first to explore the dose of RSL3 to be applied in vivo. The results demonstrated that RSL3 at 250 mg/kg was the lowest dose that effectively attenuated survival (Figure 2A). Therefore, this study explored the role of APAP in the GPX4 pathway using RSL3 250 mg/kg.

RSL3 did not significantly reverse APAP‐induced survival in mice. The results of the survival experiment showed that, compared with the sham group, the survival rate of the CLP group was significantly reduced (*p* <0 .05 vs. the sham group), and there was no significant difference in the sham+APAP group (*p* >0 .05). Compared to the CLP group, the survival rate of the APAP+CLP group was significantly higher (*p* <0 .05), and there was no significant difference between the RSL3+CLP group and the APAP+CLP+RSL3 group (*p* > 0.05). Compared with the APAP+CLP group, there was no significant difference in the survival rate of the APAP+RSL3+CLP group (*p* >0 .05) (Figure 2B).

**FIGURE 3:**
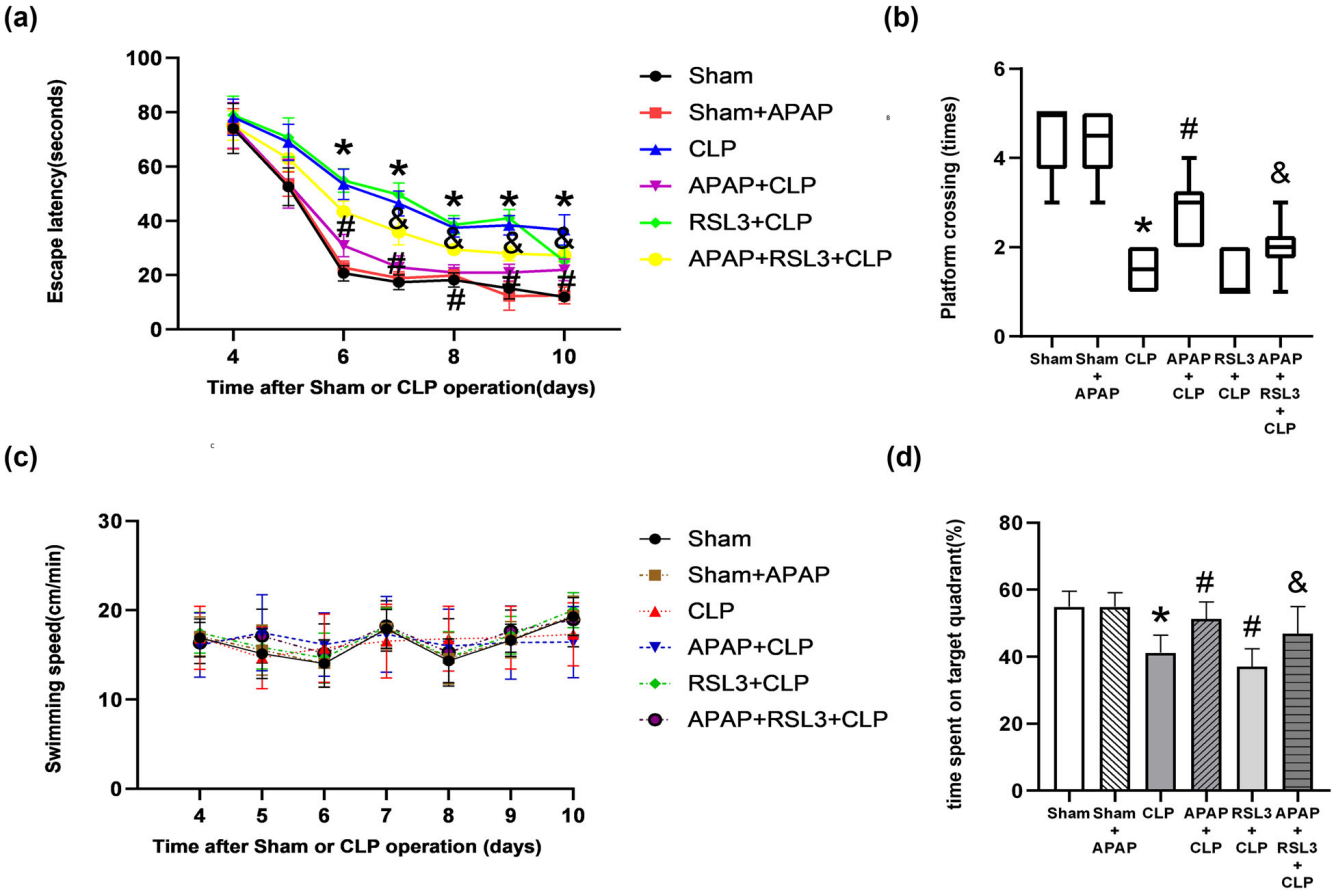
RSL3 reverses the improved cognitive function of septic mice with APAP treatment. (A–C) Escape latency, (D) platform crossing times, (E) swimming speed, and (F) time spent in the target quadrant were detected in each group (*n* = 10). *p <0 .05 versus sham group; ^#^ *p* <0 .05 versus CLP group; ^&^ *p* <0 .05 versus APAP+CLP group.

RSL3 reverses the effect of APAP on cognitive dysfunction in septic mice. The MWM results showed that compared to the sham group, the escape latency of the CLP group was significantly longer (*p* < 0.05 vs. sham group); there was no significant difference in the sham+APAP group (*p* > 0.05 vs. sham group). Compared with the CLP group, the escape latency of the APAP+CLP group was significantly reduced (*p* < 0.05); there was no significant difference in the RSL3+CLP group and APAP+CLP+RSL3 group (*p* >0 .05). Compared to the APAP+CLP group, escape latency was significantly longer (*p* < 0.05 vs. the APAP+CLP group) (Figure 3A). Compared with the sham group, the time spent in the target quadrant and platform crossing times were significantly reduced in the CLP group (*p* <0 .05, sham group); both were not significantly different in the sham+APAP group (*p* >0 .05 vs. sham group). Compared with the CLP group, the APAP+CLP group showed significantly increased platform crossing times and time spent in the target quadrant (*p* <0 .05); the RSL3+CLP and APAP+CLP+RSL3 groups did not have significantly changed platform crossing times (*p* >0 .05) but spent significantly more time in the target quadrant (*p* <0 .05). Compared to the APAP+CLP group, the platform crossing times and time spent in the target quadrant in the APAP+RSL3+CLP group were significantly higher (*p* <0 .05 vs. APAP+CLP group) (Figure 3B,D). There was no significant difference in swimming speed between the groups (*p* > 0.05) (Figure 3C).

RSL3 reverses the role of APAP in reducing hippocampal tissue damage. Neurons in the CA1 region of the hippocampus of the sham and sham+APAP groups were clearly structured and tightly arranged. The neurons in the CLP group were ill‐structured and disorganized, with dark cytoplasmic staining and nuclear consolidation. In the APAP+CLP group, the neurons were still orderly arranged, the majority of neurons had normal morphology, and a small number of neurons were damaged. In the RSL3+CLP group, the neurons were very sparse and ill‐structured, disorganized, with deep cytoplasmic staining and nuclear consolidation, and substantial neurons were damaged. In the APAP+RSL3+CLP group, the neurons were ill‐structured and disorganized, with deep cytoplasmic staining and nuclear consolidation, and a large number of neurons were damaged. Relative to the CLP group, the number of abnormal neurons was significantly decreased in the APAP+CLP group. The number of abnormal neurons was significantly increased in the RSL3+CLP group and APAP+CLP+RSL3 group. Relative to the APAP+CLP group, the number of abnormal neurons was significantly increased in the APAP+RSL3+CLP group (Figure 2C).

**FIGURE 4:**
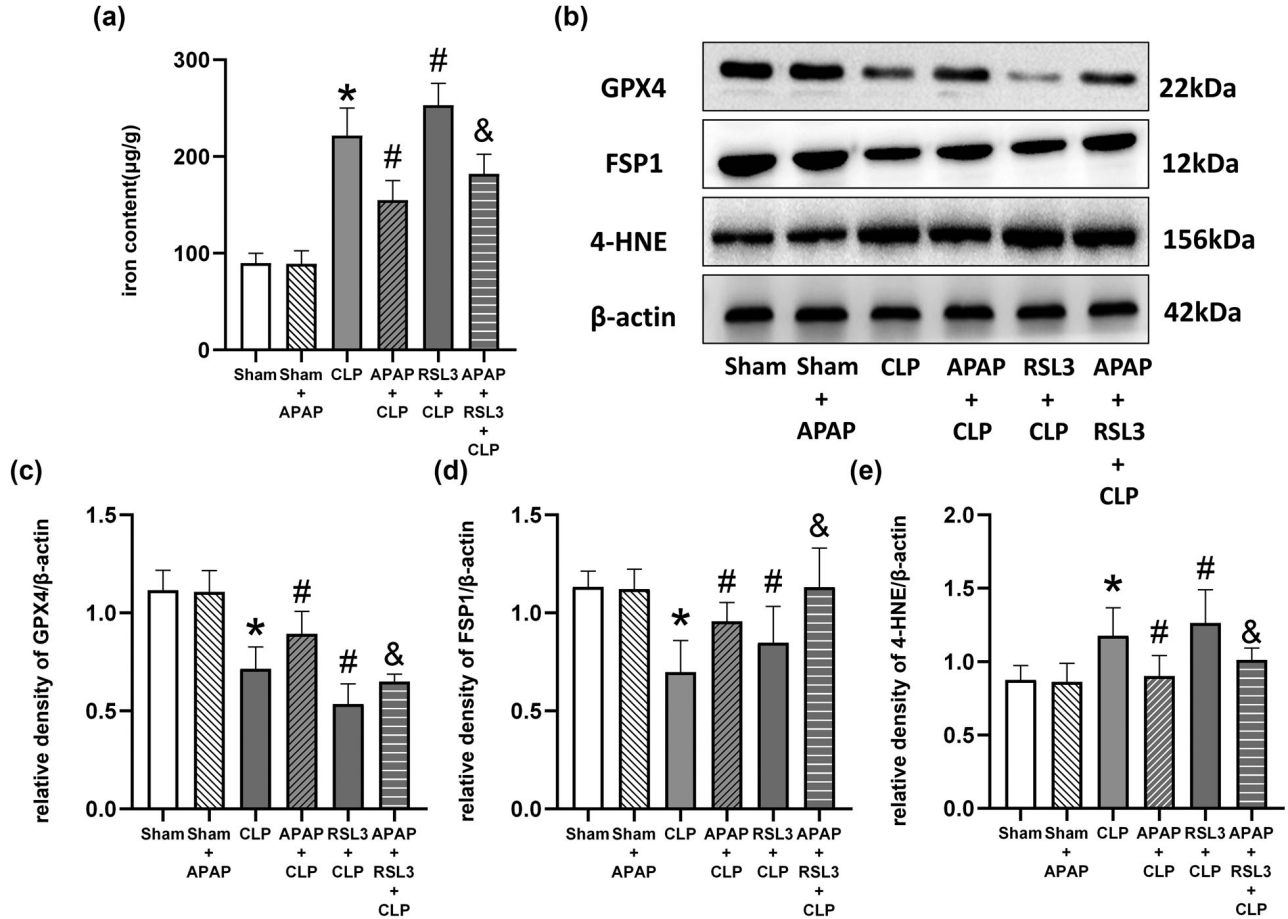
RSL3 reverses the regulation of iron content and ferroptosis marker proteins of septic mice with APAP treatment. (A) Iron content of the hippocampus was detected in each group (*n* = 6). (B) Western blot images of GPX4, FSP1, and 4‐HNE in the hippocampal tissues of mice in each group. Comparison of the quantitative analysis of GPX4 (C), FSP1 (D), and 4‐HNE (E) in the hippocampal tissues of mice in each group. *p <0 .05 versus sham group; ^#^ *p* < 0.05 versus CLP group. ^&^ *p* < 0.05 versus APAP+CLP group.

Western blotting showed that RSL3 altered the role of APAP in the regulation of ferroptosis marker proteins in mice with sepsis. In contrast to the sham group, the expression of GPX4 and FSP1 was significantly reduced, and the expression of 4‐HNE was significantly elevated in the CLP group (*p* <0 .05); the expression of GPX4, FSP1, and 4‐HNE was not significantly different in the sham+APAP group (*p* >0 .05). In contrast to the CLP group, APAP treatment elevated the expression of GPX4 and FSP1 while reducing the expression of 4‐HNE in the APAP+CLP group (*p* <0 .05 vs. CLP group). In the RSL3+CLP group, the GPX4 level was significantly reduced (*p* <0 .05 vs. CLP group), while FSP1 and 4‐HNE levels were significantly elevated (*p* >0 .05 vs. CLP group). In the APAP+CLP+RSL3 group, the FSP1 level was significantly elevated (*p* <0 .05 vs. CLP group), but the GPX4 and 4‐HNE levels were significantly changed (*p* > 0.05 vs. CLP group). In contrast to the APAP+CLP group, the GPX4 levels were significantly reduced in the APAP+RSL3+CLP group, while the FSP1 and 4‐HNE levels were significantly elevated (*p* <0.05 vs. APAP+CLP group) (Figure 4B–E).

**FIGURE 5:**
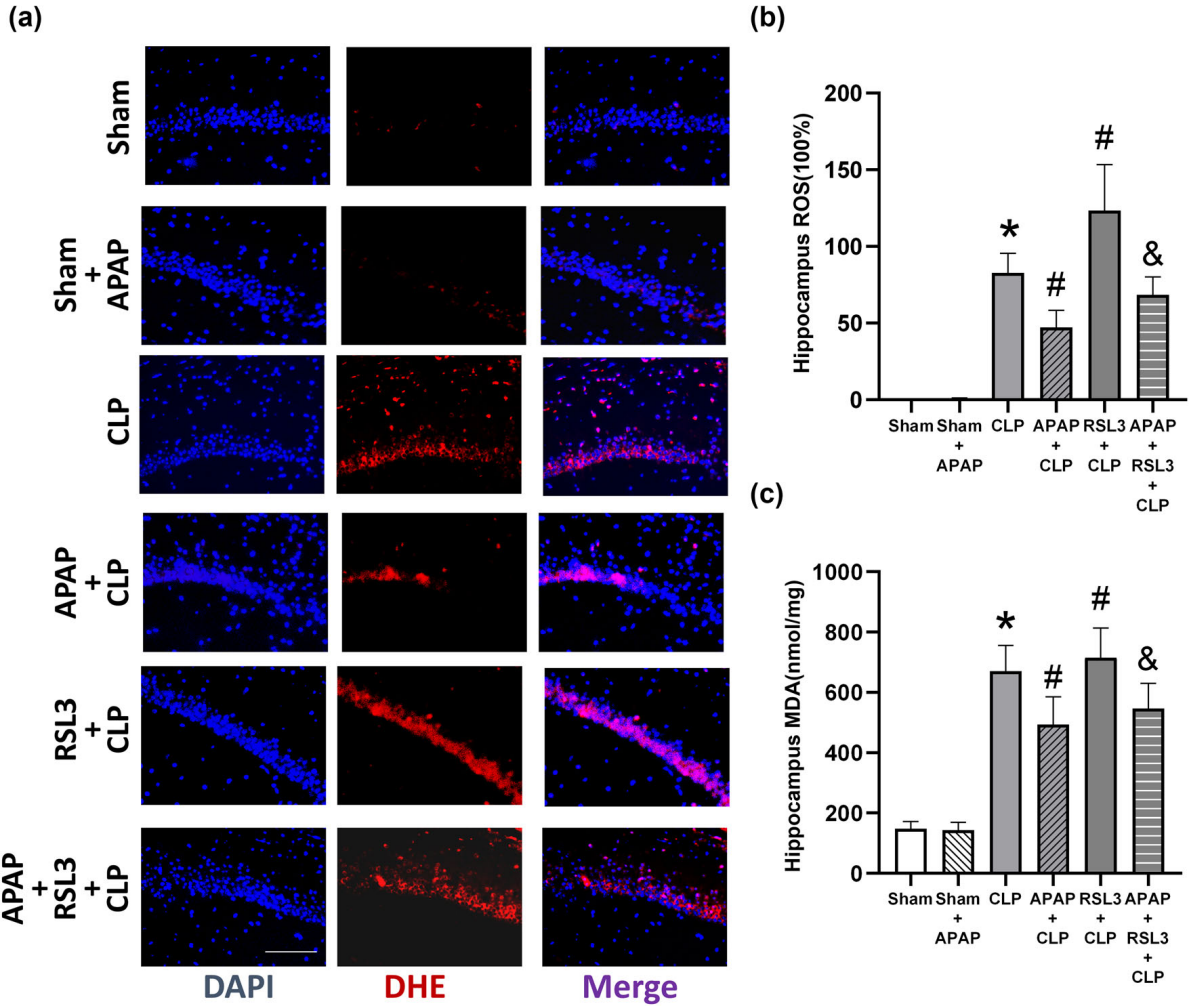
RSL3 reverses the regulation of ROS and MDA levels of septic mice with APAP treatment. (A) Immunofluorescence staining of ROS of the hippocampus in septic mice. (B) Quantitative analysis of ROS of the hippocampus in septic mice is shown as the relative density to that of the sham group (*n* = 6). The ratio of the sham group was defined as 100%. (C) MDA content of the hippocampus was detected in each group (*n* = 6). *p <0 .05 versus sham group; ^#^ *p* <0 .05 versus CLP group; ^&^ *p* <0 .05 versus APAP+CLP group.

RSL3 also reversed the effects of APAP on iron, ROS, and MDA levels in the cerebral hippocampus. In contrast to the sham group, iron, MDA, and ROS levels were significantly elevated in the CLP group (*p* <0 .05). However, iron, MDA, and ROS levels were not significantly different in the sham+APAP group (*p* > .05). In contrast to the CLP group, APAP treatment reduced iron, MDA, and ROS levels in the APAP+CLP group (*p* <0 .05). In the RSL3+CLP group, iron, MDA, and ROS levels were significantly elevated (*p* < 0.05). In the APAP+CLP+RSL3 group, iron, MDA, and ROS levels were not significantly elevated (*p* >0 .05). Iron, MDA, and ROS levels were significantly elevated in the APAP+RSL3+CLP group compared to the APAP+CLP group (*p* < 0.05 vs. APAP+CLP group) (Figures 4A and 5).

### 3.2 iFSP1+RSL3 further reversed the inhibitory effect of APAP on ferroptosis in the cerebral hippocampus of septic mice

**FIGURE 6:**
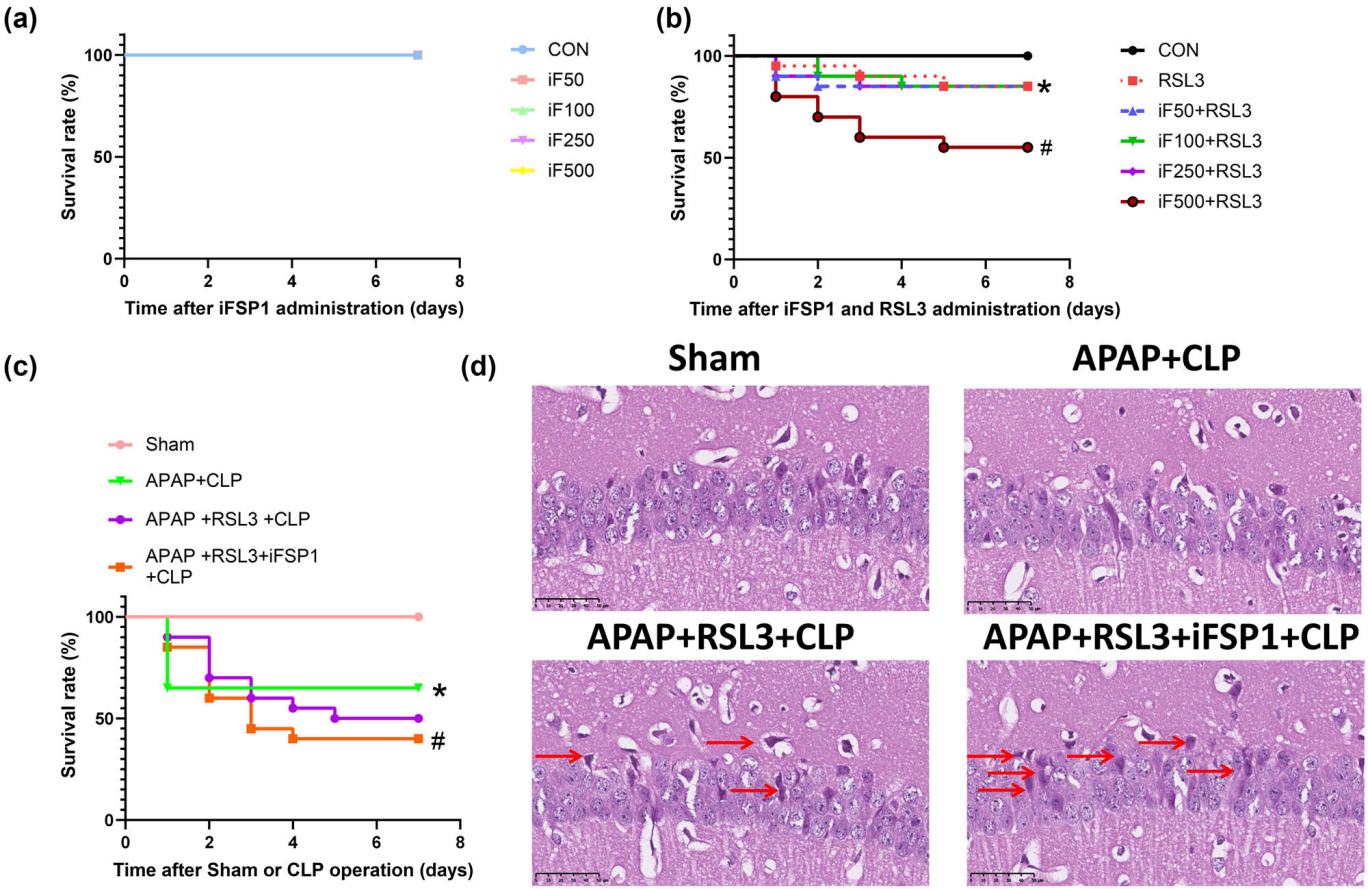
Effect of different doses of iFSP1 on the 7‐day survival rate of healthy mice (A). Effect of different doses of iFSP1 combined with RSL3 on the 7‐day survival rate of healthy mice (B). iFSP1 significantly reverses the effect of survival boosted by APAP but not RSL3 (C). Compared to RSL3, iFSP1+RSL3 further reversed the improved hippocampi damage of septic mice with APAP treatment (D). (A–C) Values are depicted as the survival percentage (*n* = 20). *p <0 .05 versus CON group; ^#^ *p* <0 .05 versus RSL3 group (B). *p <0 .05 versus sham group; ^#^ *p* <0 .05 versus APAP+CLP group (C). (D) Brain tissue sections were made and stained with hematoxylin and eosin. The hippocampi structure was observed.

In the present study, we explored the application of iFSP1 in vivo. The results demonstrated no significant difference in the survival rate of mice with iFSP1 50–500 mg/kg (*p* >0 .05) (Figure 6A). These results were completely different when iFSP1 was administered in combination with RSL3 (250 mg/kg). In contrast to the RSL3 group, the survival rate was significantly reduced in the iF500+RSL3 group (*p* <0 .05 vs. RSL3 group) (Figure 6B). Therefore, 500 mg/kg iFSP1 combined with 250 mg/kg RSL3 was selected for the intervention.

The results of the survival experiments showed that iFSP1+RSL3 successfully reversed the APAP‐induced survival rate. The survival rate was significantly lower in the APAP+CLP group than in the sham group (*p* <0 .05). In contrast to the APAP+CLP group, there was no significant difference in the survival rate in the APAP+RSL3+CLP group (*p* > 0.05 vs. APAP+CLP group). However, the survival rate was significantly reduced in the APAP+RSL3+iFSP1+CLP group (*p* < 0.05 vs. APAP+CLP group) (Figure 6C).

**FIGURE 7:**
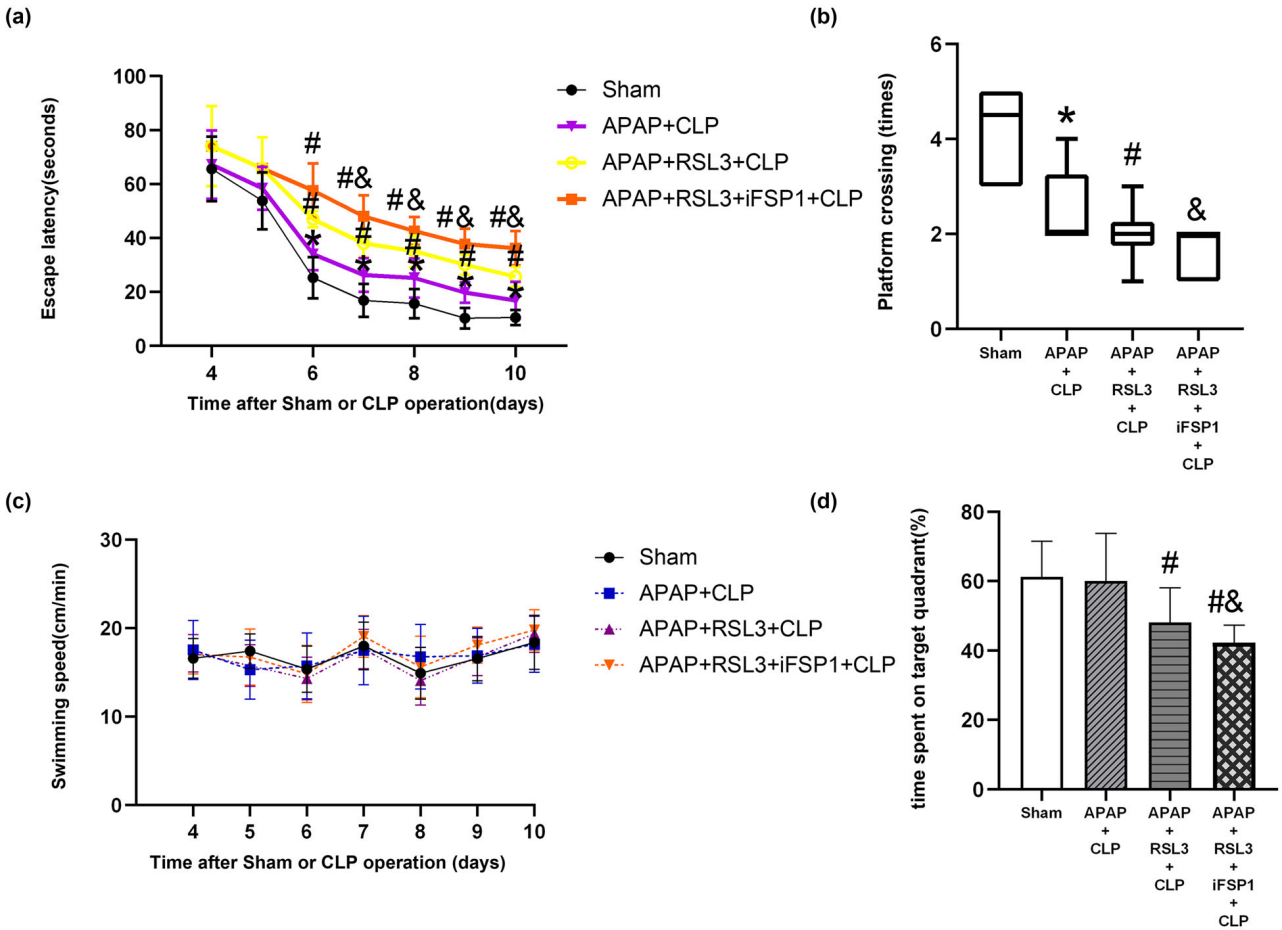
Compared to RSL3, iFSP1+RSL3 further reverses the improved cognitive function of septic mice with APAP treatment. (A–C) Escape latency, (D) platform crossing times, (E) swimming speed, and (F) time spent in the target quadrant were detected in each group (*n* = 10). *p < 0.05 versus sham group; ^#^ *p* <0 .05 versus APAP+CLP group; ^&^ *p* < 0.05 versus APAP+RSL3+CLP group.

iFSP1 reverses APAP‐induced cognitive dysfunction. The MWM results showed a significantly longer escape latency in the APAP+CLP group than in the sham group (*p* < 0.05). The escape latency was significantly prolonged in the APAP+RSL3+CLP and APAP+RSL3+iFSP1+CLP groups compared to that in the APAP+CLP group (*p* <0 .05). The escape latency was significantly prolonged in the APAP+RSL3+iFSP1+CLP group in contrast to the APAP+RSL3+CLP group (*p* <0 .05 vs. APAP+RSL3+CLP group) (Figure 7A). Compared to the sham group, the platform crossing times in the APAP+CLP group were significantly reduced (*p* <0 .05 vs. sham group), while the time spent in the target quadrant was not significantly changed (*p* >0 .05 vs. sham group). The number of platform crossings and time spent in the target quadrant were significantly reduced in the APAP+RSL3+CLP and APAP+RSL3+iFSP1+CLP groups compared to the APAP+CLP group (*p* <0 .05). In contrast to the APAP+RSL3+CLP group, the platform crossing times and time spent in the target quadrant were significantly reduced in the APAP+RSL3+iFSP1+CLP group (*p* < 0.05 vs. APAP+RSL3+CLP group) (Figure 7B,D). There was no significant difference in the swimming speed between groups (*p* >0 .05) (Figure 7C).

Pathological examination revealed that iFSP1+RSL3 reversed the effects of APAP on reducing hippocampal tissue damage. The neurons of the hippocampal CA1 region in the sham group were clearly structured and closely arranged. The neurons in the APAP+CLP group were still orderly arranged, the majority had normal morphology, and a few were damaged. The neurons in the APAP+RSL3+CLP group were sparse and structurally indistinct, accompanied by disorganized arrangement, deep cytoplasmic staining, and nuclear consolidation; the neurons in the APAP+RSL3+iFSP1+CLP group were sparse, with extremely indistinct structure, extremely disorganized arrangement, deep cytoplasmic staining, and increased nuclear consolidation. In contrast to the sham group, the number of abnormal neurons in the CA1 region was significantly higher in the APAP+CLP group. In contrast to the APAP+CLP group, the number of abnormal neurons in the APAP+RSL3+CLP and APAP+RSL3+iFSP1+CLP groups significantly increased. In contrast to the APAP+RSL3+CLP group, the number of abnormal neurons in the APAP+RSL3+iFSP1+CLP group significantly increased (Figure 6D).

**FIGURE 8:**
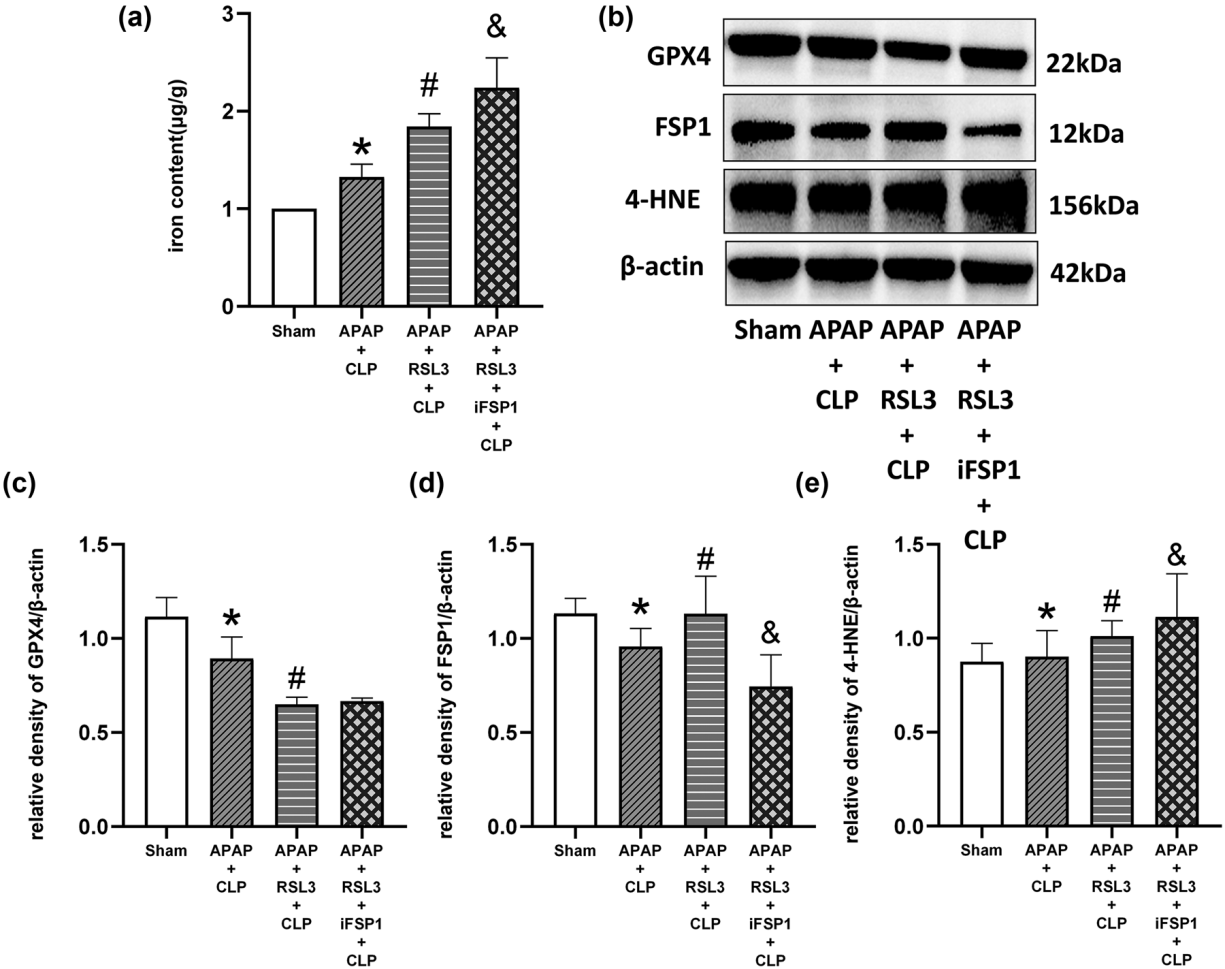
Compared to RSL3, iFSP1+RSL3 further reverses the regulation of iron content and ferroptosis marker proteins of septic mice with APAP treatment. (A) Iron content of the hippocampus was detected in each group (*n* = 6). (B)Western blot images of GPX4, FSP1, and 4‐HNE in the hippocampal tissues of mice in each group. Comparison of the quantitative analysis of GPX4 (C), FSP1 (D), and 4‐HNE (E) in the hippocampal tissues of mice in each group. *p <0 .05 versus sham group; ^#^ *p* <0 .05 versus APAP+CLP group; ^&^ *p* <0 .05 versus APAP+RSL3+CLP group.

Western blot results showed that iFSP1+RSL3 further reversed the role of APAP in the regulation of ferroptosis marker proteins in the cerebral hippocampus. In contrast to the sham group, the GPX4 and FSP1 levels were significantly decreased, while the 4‐HNE levels were elevated in the APAP+CLP group (*p* <0 .05, sham group). As an inhibitor of GPX4, RSL3 significantly decreased GPX4 expression and increased 4‐HNE expression but elevated FSP1 expression in the APAP+RSL3+CLP group, in contrast to the APAP+CLP group (*p* <0 .05). iFSP1 inhibited the elevated expression of FSP1. In the APAP+RSL3+iFSP1+CLP group, the GPX4 and FSP1 levels were significantly decreased, while the 4‐HNE levels were elevated in contrast to the APAP+CLP group (*p* <0 .05). In the APAP+RSL3+iFSP1+CLP group, FSP1 was further decreased, and the 4‐HNE levels were further elevated (*p* <0 .05 vs. APAP+RSL3+CLP group), while the expression of GPX4 was not significantly changed compared to the APAP+RSL3+CLP group (*p* >0 .05 vs. APAP+RSL3+CLP group) (Figure 8B–E).

**FIGURE 9:**
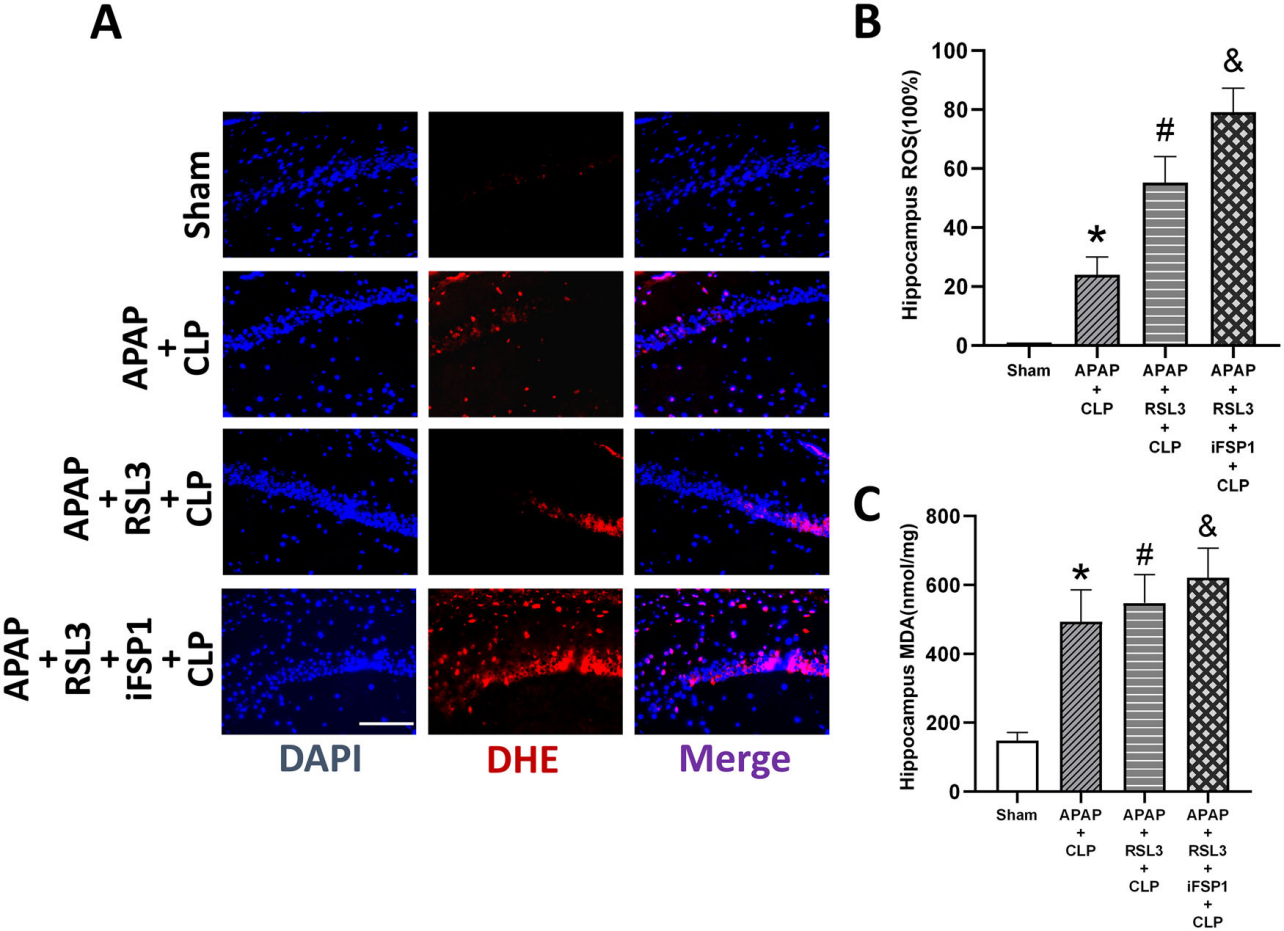
Compared to RSL3, iFSP1+RSL3 further reverses the regulation of ROS and MDA levels of septic mice with APAP treatment. (A) Immunofluorescence staining of ROS of the hippocampus in septic mice. (B) Quantitative analysis of ROS of the hippocampus in septic mice is shown as the relative density to that of the sham group (*n* = 6). The ratio sham group was defined as 100%. (C) MDA content of the hippocampus was detected in each group (*n* = 6). *p <0 .05 versus sham group; ^#^ *p* <0 .05 versus APAP+CLP group; ^&^ *p* <0 .05 versus APAP+RSL3+CLP group.

iFSP1+RSL3 further reversed the effect of APAP on iron, ROS, and MDA levels in the cerebral hippocampus. Iron, MDA, and ROS levels were significantly higher in the APAP+CLP group than in the sham group (*p* <0 .05). In contrast to the APAP+CLP group, iron, MDA, and ROS levels were significantly higher in the APAP+RSL3+CLP and APAP+RSL3+iFSP1+CLP groups (*p* <0 .05). In contrast to the APAP+RSL3+CLP group, iron, MDA, and ROS levels were significantly higher in the APAP+RSL3+iFSP1+CLP group (*p* <0 .05 vs. APAP+RSL3+CLP group) (Figures 8A and 9).

## 4 DISCUSSION

Few clinical investigations of sepsis therapies using low‐dose APAP have been documented, and reports on its mechanisms are contentious. Clinical studies have shown that a modest dose of APAP reduces oxidative damage in patients with sepsis by reducing oxidative stress (Janz et al., 2015). However, other research has shown that the antioxidant effect of APAP is caused by its antipyretic activity (Patanwala et al., 2018). It has been stated that APAP has great potential as a traditional anti‐inflammatory and antioxidant drug for the treatment of sepsis and that further research into the molecular mechanisms and biochemical responses of APAP in the treatment of sepsis is required to demonstrate its beneficial effects on clinical treatment and to better guide its clinical application in the future (Husain & Martin, 2015).

SAE is a global concern. Neuronal injury and impairment are significantly linked to cognitive dysfunction (Ngwenya & Danzer, 2018). In recent years, there has been growing evidence that ferroptosis contributes to neuronal cell death and causes brain dysfunction in SAE (Abbah et al., 2022; Wu et al., 2022). One of our previous studies found that low‐dose APAP reduced cognitive impairment and enhanced survival in septic mice; therefore, we first examined the GPX4 pathway mechanism in vitro (Chu et al., 2022). However, the specific mechanism through which APAP inhibits ferroptosis in vivo has not yet been elucidated.

For the first time, APAP was reported to prevent ferroptosis in septic mice via the GPX4/FSP1 pathway. Ferroptosis is characterized by lipid peroxidation followed by the formation of a substantial quantity of ROS. We discovered that APAP therapy decreased hippocampal tissue damage and cognitive impairment, increased the survival rate of septic mice, and dramatically reduced iron content, 4‐HNE, and ROS expression in the hippocampal tissue of septic mice. This implies that APAP reduces ferroptosis in the hippocampal tissues of septic mice. Both GPX4 and FSP1 expression were considerably enhanced following the administration of APAP in the ferroptosis pathway protein test, showing that the prevention of ferroptosis by APAP in the hippocampal tissue of septic mice was associated with both the GPX4 and FSP1 pathways.

To confirm whether APAP inhibits hippocampal tissue ferroptosis in septic mice through the GPX4 pathway, we used a GPX4 pathway inhibitor, RSL3. In APAP‐treated septic mice, RSL3 treatment inhibited GPX4, increased FSP1 expression, and significantly reversed APAP‐reduced iron and ROS content, lipid peroxidation indicators 4‐HNE and MDA, and APAP‐mitigated cognitive dysfunction; however, survival in APAP‐boosted mice was not significantly reduced. This implies that APAP reduced ferroptosis via the GPX4 pathway. In contrast, increased FSP1 expression, as well as the nonsignificant survival rate reversal, suggests that FSP1 acts as a compensating factor when GPX4 is blocked to supplement the antilipid oxidation effect.

To demonstrate whether APAP also inhibits ferroptosis via the FSP1 pathway, studies were conducted using the FSP1 inhibitor iFSP1. iFSP1 is a selective FSP1 inhibitor that is frequently used in ferroptosis research (Jo et al., 2022). The survival rate of mice remained unaltered when iFSP1 alone was administered up to 500 mg/kg in a dose‐dependent manner, indicating that GPX4 produces a substantial antilipid peroxidation effect in healthy mice, which is adequate to compensate for the decreased redox function of FSP1. In contrast, when mice were administered 500 mg/kg of iFSP1 in addition to RSL3, their survival rate was much lower than when RSL3 was administered alone, indicating that the combined action of the GPX4 and FSP1 pathways reduced ferroptosis in healthy mice. Consequently, during the subsequent round of route discovery, researchers chose a combination of iFSP1 and RSL3 pathway inhibitors. These results demonstrated that when iFSP1 was added indefinitely, FSP1 was suppressed. iFSP1+RSL3 resulted in a substantial reversal of iron and ROS content, lipid peroxidation markers 4‐HNE and MDA, and cognitive impairment, which was mitigated by APAP compared to RSL3 alone. Furthermore, iFSP1+RSL3 treatment drastically reversed the survival rate of APAP‐treated mice. These findings show that APAP inhibits ferroptosis in mice with sepsis via the GPX4 and FSP1 pathways.

The ferroptosis pathways has not yet been fully elucidated, but GPX4 and FSP1 were the most important roles. Among the identified ferroptosis pathways, the xCT/GSH/GPX4 axis is the predominant negative regulatory system (Ursini & Maiorino, 2020). GPX4, the most important downstream regulator of this system, was the first identified inhibitor of central ferroptosis (Stockwell et al., 2017). It converts GSH to GSSG and reduces cytotoxic lipid peroxides to their corresponding alcohols by depleting nicotinamide adenine dinucleotide phosphate (NADPH/H^+^). GPX4 plays an important role in the inhibition of lipid peroxidation in subcellular structures, such as the cytoplasmic membrane, mitochondria, and nucleus, and is of great importance in the inhibition of cellular lipid peroxidation.

FSP1 is the most important negative regulator of ferroptosis other than GPX4. FSP1 parallels the GPX4 system and relies on NADPH/H^+^ mainly to promote the reduction of ubiquinone (CoQ_10_) to reduce Coenzyme Q (CoQH_2_) (which directly reduces lipid peroxide radicals to terminate lipid autoxidation) on the plasma membrane, or indirectly by promoting the regeneration of α‐tocopherol (vitamin E, a powerful natural antioxidant) to antagonize Fe^2+^‐mediated lipid peroxidation and reduce cellular ferroptosis (Bersuker et al., 2019; Doll et al., 2019). FSP1 plays an important role in antilipid oxidation in the cytoplasmic membrane.

Previous studies have shown that small doses of APAP exert neuroprotective effects through nonenzymatic antioxidant systems, including the modulation of GSH‐Px enzyme activity and vitamin E (α‐tocopherol) levels (Ghanem et al., 2016). This pathway is closely related to the GPX4 and FSP1 pathways. Our study demonstrated that APAP inhibited ferroptosis in the cerebral hippocampus of septic mice through GPX4 combined with the FSP1 pathway.

Understanding the specific molecular mechanisms by which APAP inhibits ferroptosis in the cerebral hippocampus of septic mice is essential for understanding the sites and mechanisms through which APAP exerts its neuroprotective effects and will be an important guide for future clinical work. This study contributes to understanding the molecular mechanisms by which APAP inhibits ferroptosis. Further studies are needed to elucidate whether APAP exerts its ferroptosis inhibitory effects through other ferroptosis pathways, especially the relationship between APAP and mitochondrial ferroptosis.

## 5 CONCLUSION

APAP inhibits ferroptosis in the cerebral hippocampus of septic mice through the GPX4 and FSP1 pathways.

## AUTHOR CONTRIBUTIONS

**Jing Chu**: Methodology, Software, Data curation, Writing–original draft. **Hong Li**: Methodology, Data curation. **Zhihao Yuan**: Data curation, Software development. **Wenyu Zhou**: Software, Validation. **Yang Yu**: Conceptualization, Writing–review, and editing. **Yonghao Yu**: Supervision.

### PEER REVIEW

The peer review history for this article is available at https://publons.com/publon/10.1002/brb3.3145

## Data Availability

The datasets used and analyzed in the present research are available from the corresponding author upon reasonable request.
